# Feasibility Study of Using Hydrophobic Geopolymer-Based as Aggregate Substitution in Asphalt Mixture

**DOI:** 10.3390/polym15143077

**Published:** 2023-07-18

**Authors:** Cadnel Ago, Guowei Li, Jiantao Wu, Nur Izzi Md Yusoff

**Affiliations:** 1 College of Civil and Transportation Engineering, Hohai University, Nanjing 210098, China; cadkvic@hotmail.com (C.A.); lgwnj@163.com (G.L.); 2 Department of Civil Engineering, Universiti Kebangsaan Malaysia, Bangi 43600, Malaysia

**Keywords:** hydrophobic aggregate, asphalt, geopolymer, artificial aggregate, alkali-silicate activation

## Abstract

Hydrophobic aggregates have the great ability to prevent asphalt pavement roads from stripping-off of the asphalt in presence of water. In addition, they give the option to consume less asphalt and save cost. On the other hand, natural aggregates have been found to be non-renewable and rare. Geopolymer based artificial aggregates are great materials as they demonstrated to have exceptional features, such as high strength, superior durability, and greater resistance to fire exposure. In this study, a new hydrophobic geopolymer based aggregate has been produced with rice ash (RA) and fly ash as precursors as well as, Sodium Hydroxide (NaOH) and Sodium Silicate (Na_2_SiO_3_) as activators. The mechanical properties combined with the softening coefficient, surface properties of samples, contact angle and adhesion were characterized as well as microstructure X-ray diffraction (XRD) and Scanning electron microscopy (SEM) test. The results indicate that the activators Na_2_SiO_3_/NaOH at a mix ratio of 1 have a suitable effect on the pores and the compressive strength of the new artificial aggregate most particularly sodium hydroxide. Nonetheless, it has been found that coating the artificial aggregate with asphalt showed a great improvement of the hydrophobic nature of the produced artificial aggregate based geopolymer. Hence, indicates the possibility of using it as recycle aggregate pavement. From a microstructure point, the hydrophobic nature of the new alkali-activated artificial aggregate can be improved by increasing the quantity of mullite in the mix proportion design.

## 1. Introduction

In terms of strength, corrosion resistance, and sealing ability, hydrophobic geopolymer-based artificial aggregate, a novel kind of green cementitious material, excels, making them a preferred option for use in pavement, building, and aerospace materials [1,2]. Fly ash is a common reusable material that is produced when coal is burned [3]. Due to the distinctive function that fly ash possesses as a result of its ball-bearing structure, which makes it valuable in the production of sustainable materials, fly ash is a perfect precursor [4]. Furthermore, both fly ash and rice ash have been acknowledged as pozzolanic geopolymerization components and have shown remarkable performance in mechanical property, durability, and usable bonding capacity [5,6].

The study of geopolymers has piqued the interest of many professionals in related domains. The initial component used in the synthesis of geopolymers was kaolinite [7], and later geopolymers were made from a variety of basic waste materials [8,9]. Others looked into the integration of polymers since they encourage reuse and recycling while preserving sources and lowering costs [10,11]. The majority of these studies concentrate on their uses in polymers, soil stabilization, sustainable concrete, geopolymer concrete, and aggregate alternatives. A study on the impact of fly-ash-based geopolymer coated aggregate on bituminous mixtures [12] in the context of asphalt pavement came to the conclusion that it contributes to greater stability and longer service life in bituminous mixtures.

Moreover, using composites modified with hydrophobic particles and polydimethylsiloxane, a waterproof geopolymer study was conducted [13] which provided guidance on how to waterproof engineered geopolymer materials. Furthermore, the development of reclaimed asphalt pavement (RAP) pervious concretes has prompted research into the chemical, strength, adhesion, and morphological characteristics of geopolymer-modified asphalt based on fly ash [14,15,16]. Additionally, a study was conducted to determine the effects of diffusion between virgin and aged asphalt binders; asphalt pavements generally consume a significant amount of asphalt, which raises the project cost, especially in terms of absorption and the necessary binder content. Hence, the ideal amount of asphalt binder being an important subject, leaded to the need of studying the improvement of Water Resistance of Asphalt Concrete Wearing Course Using Latex-Asphalt Binder [16,17].

The work done in this present study, aims to evaluate the hydrophobic nature of a new designed artificial aggregate based geopolymer as aggregate replacement in asphalt pavement mixtures.

## 2. Materials and Methods

### 2.1. Materials

The Fly Ash class C used in this study is a commercial product, similar for the activators Alkali-Silica composed of Sodium Hydroxide (NaOH) and an 8 M Sodium Silicate (Na_2_SiO_3_). Table 1 shows the main chemical parameters of the raw materials analyzed by XRD technique via an X-ray diffractometer (RIGAKU, model D/max) in advance. The asphalt used in this study is a commercial penetration grade (Pen) 70 asphalt (Table 2). A waste recycled concrete with a compressive strength of 40 kN/mm was used for comparison. The Polycarboxylate (PCE) is a 40% solid content liquid form commercial Superplasticizer. The rice ash used in this study is also a commercial admixture.

### 2.2. Experimental Methods

This present work was done in two principal sections.

Section 1: design and production of strong and resistant artificial aggregate based geopolymer. For this purpose, rice ash has been added to the mixture specifically as sand replacement.

Section 2: hydrophobisation of the produced geopolymer based artificial aggregate. For this purpose, the produced aggregates were crushed in small sizes and sieved in coarse and fine aggregates and were coated with asphalt.

**Figure 1:**
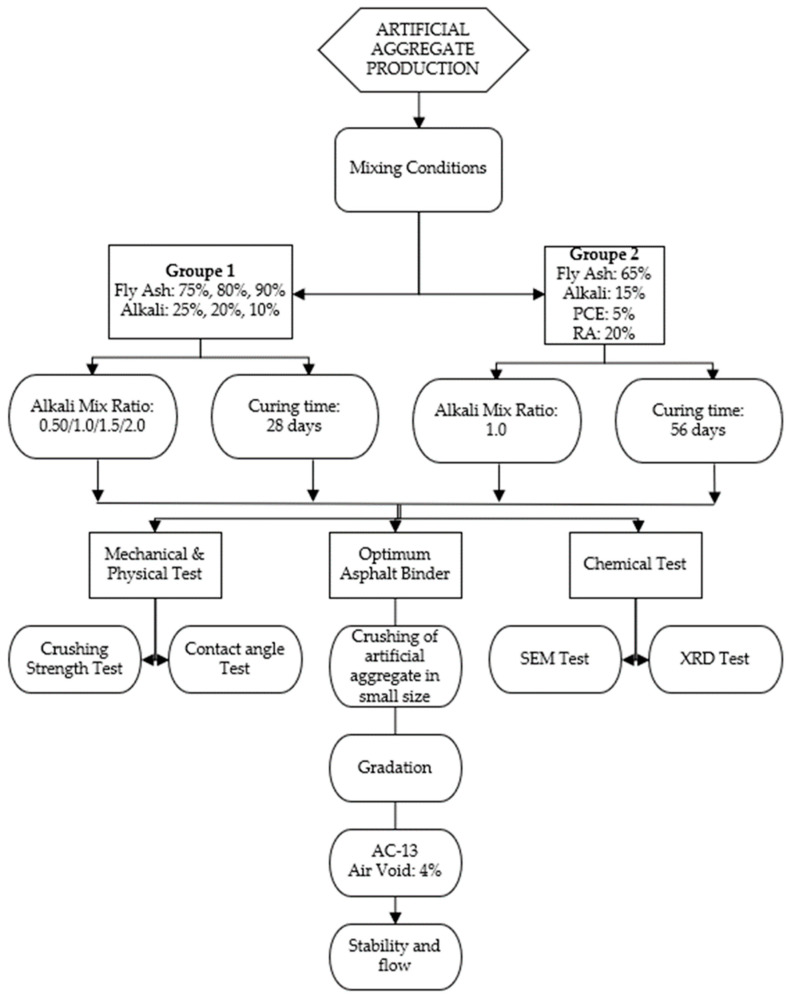
Experiment’s flow chart.

Figure 1 gives a small description of the whole experiment procedure followed in this present work and Table 3 describes the different experiment steps.

As described in Figure 1, the experiment in this present work was done in two groups. In the first group (group 1), several specimens were executed without addition of RA in opposition to the second group (group 2), all the specimens were done with addition of RA as comparison for the compressive strength and addition of PCE in order to reduce the use of alkali (Na_2_SiO_3_/NaOH) in the mixture design. Each mix proportion were prepared by partial percent replacement of the total mixture’s weight. To prepare well the mortar paste, the Sodium Hydroxide (NaOH) and Sodium Silicate (Na_2_SiO_3_) activators were used at different liquid to binder mass ratio 0.50/1.0/1.5/2.0 for group 1 and for the group 2, only a liquid to binder mass ratio 1.0 were used.

The mix proportions used to test both the compressive strength and the water absorption are described in Table 3.

The specimens were casted in 15 cm × 15 cm × 15 cm cubical molds and were cured for periods of 28 days and 90 days. At first, the specimens were cured at room temperature for 10 days, demolded and inserted in an oven at a temperature of 90 °C for 10 days and finally at room temperature for the remaining days. The mechanical properties have been tested according to the International Standard of Aggregates for concrete: Test methods for mechanical and physical properties (BS EN 1097) [21] including the compressive strength test and the Los Angeles Abrasion test.

**Figure 2:**
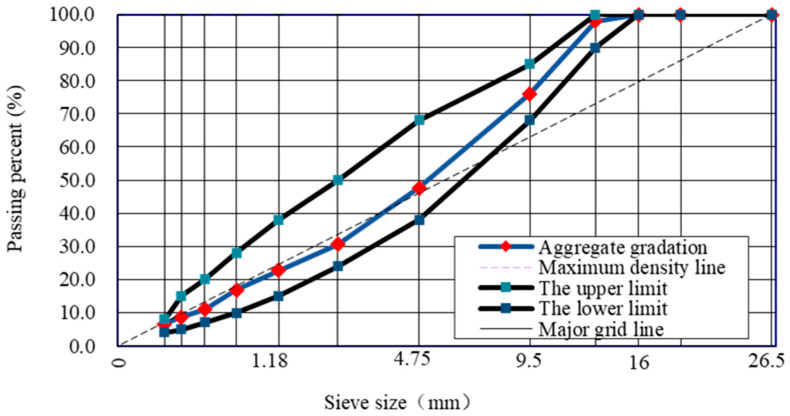
Design of aggregate gradation size.

Since the specimens of group 2 were found to give a better balance of the activators Na_2_SiO_3_/NaOH in the mix design (Figure 2), only the specimens of the group 2 have been used for the whole study. In this present study, several key hydrophobic properties have been followed in order to characterize and evaluate well the hydrophobic nature of the new artificial aggregate based geopolymer. Among these key properties can be cited: the water absorption which was determined to analyze the waterproof property. The water absorption (*W_a_*) is a parameter related to the waterproof property [22]. The softening coefficient, which represents the quality, particularly mechanical property preserved after saturating water, is an important criterion for evaluating waterproof of materials. The ratio of the compressive strength after water saturation to that after drying was defined as the softening coefficient [22]. The microhardness of the specimen’s surface which was measured to evaluate the compactness of the specimen’s surface [23]. The contact angle and adhesion which was measured by using the contact angle goniometer (SDC-100S), a system with a maximum error was 0.1°. The liquid droplet was deposited under gravity by a syringe pointed vertically down onto the surface of specimen. A high-resolution camera might record the angle and analysis software could examine it [24]. Using a micropipette, uniform liquid drop volumes of around 5 mm^3^ were administered to the sample surface. The surfaces of samples were corrected smoothed to mitigate the impact of surface roughness. Five drops were applied onto each sample, given the heterogeneity of the material. The measurements were conducted at the temperature approximating 22.5 °C.

The optimum binder content has been determined following the ASTM standard D6927–15 [25]. This standard is specifically used as Test Method for Marshall Stability and Flow of Asphalt Mixtures. In this work, the gradation and asphalt mixture concrete used is AC-13 and Figure 2 shows the gradation that has been followed. In this work, different compacted specimens at different trial asphalt content were done with an increment of 0.5 percent binder content on a range from 3.5 to 5.5 percent binder content. In total 15 specimens were done and measured.

## 3. Results and Discussion

### 3.1. Mechanical Performance

**Figure 3:**
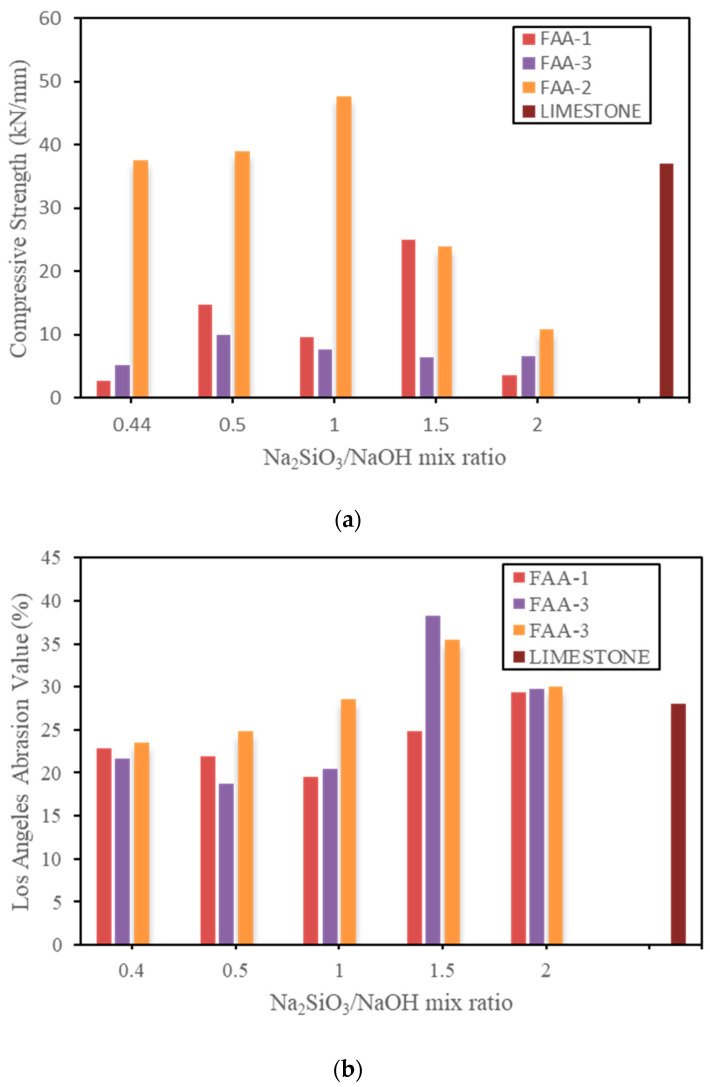
Results of mechanical properties (group 1): (**a**) Compressive strength and (**b**) Los Angeles Abrasion. Note: FAA-1 refers to the mix proportion of 75% of fly ash and 25% of Alkali on the total mass weight; FAA-2 refers to the mix proportion of 80% of fly ash and 20% of Alkali on the total mass weight; FAA-3 refers to the mix proportion of 90% of fly ash and 10% of Alkali on the total mass weight.

**Figure 4:**
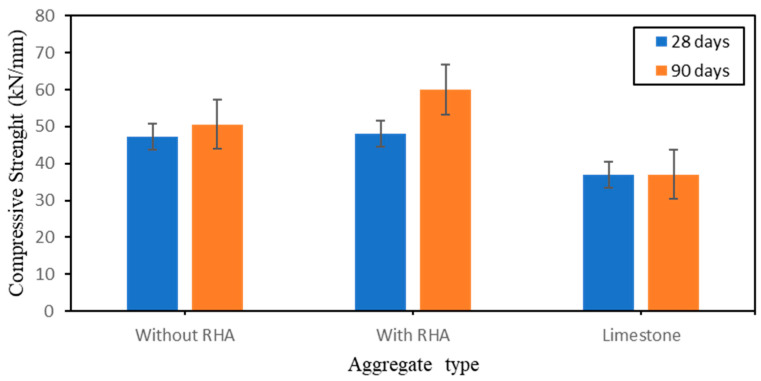
Effect of RA on the Compressive strength of aggregate type during 28 days and 90 days of curing time.

Figure 3 shows the mechanical properties of the produced artificial aggregate after 28 days of curing (group 1) and Figure 4 shows the results of the mechanical properties of the produced artificial aggregate after both 28 days and 90 days of curing time (group 2). The results obtained from the experiment shows complete varieties of values. Both alkali-Silicate Na_2_SiO_3_/NaOH mix ratio and rice ash has a strong effect on the strength of artificial aggregate. It is important to specify that (see Figure 3) Na_2_SiO_3_/NaOH mix ratio 1 leads to a good and better balance in the use of the activators sodium hydroxide (NaOH) and sodium silicate (Na_2_SiO_3_). The Results has shown that the more the curing time increases the higher the compressive strength; the reason of this effect is because the whole aggregate reach to full majority as time increase. In addition, it has been noticed that during the period of 28 days of curing the surface (outer part) of the sample is dried but the inside (inner part) of the sample is still saturated and not completely dry. Hence, after 90 days of curing time, the sample can reach a certain maturity which leads to higher strength.

Moreover, the addition of rice ash in the mix proportion has also given the possibility to have a high strength. As results from these phenomena, it was gotten after experimental curing time of 28 days and 90 days the crushing strengths of 47.3 kN/mm (without RA), 48 kN/mm (with RA) and 50.6 kN/mm (with RA), 60 kN/mm (without RA) respectively. In the same objective of testing the crushing strength, limestone has been used to as comparative natural aggregate. The compressive strengthof limestone during both 28 days and 90 days of curing time remains constant without change with a compressive strengthvalue of 37 kN/mm. Limestone aggregates, in contrast to the manufactured artificial aggregate based geopolymer, have proved to be less robust because the mineral component of natural aggregates has a lower dosage and a lower amount of strong minerals. Consequently, the ability to precisely manage the mix proportions of each resource is provided by the fabrication of artificial aggregate. (Precursors and activators). Natural aggregates, in this case limestone, cannot achieve a certain high limit of strength without particular activators or admixtures. Additionally, the region, ground, geology of the ground, and country from where natural aggregates were extracted have an impact on the composition and nature of the aggregates.

The whole analysis on the section below was done based on the results obtained from the specimens on the group 2 which is the group with the highest compressive strength value (see Figure 4).

### 3.2. Hydrophobic Properties

#### 3.2.1. Water Absorption

**Figure 5:**
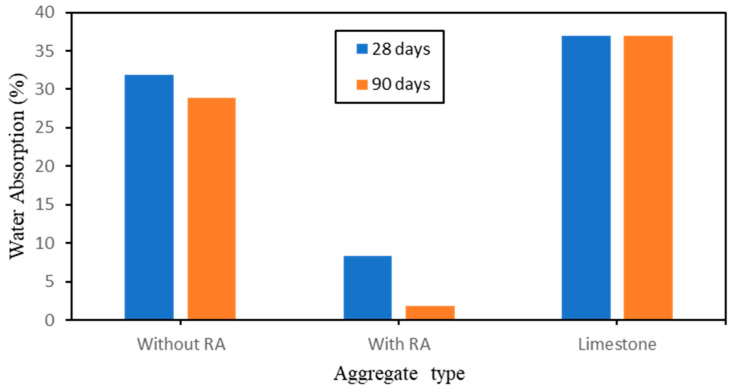
Effect of RA on the water absorption of aggregate types during 28 days and 90 days of curing time.

In order to investigate well the hydrophobic behavior of the new produced artificial aggregate, on the changes in absorptivity, multiple tests have been run. The effects of rice ash and Polycarboxylate (PCA) on the water absorption of geopolymer after 28 days and 90 days of curing age is shown in Figure 5. It overall, exhibits relative strong water absorption after 28 days of curing time and less absorption after 90 days of curing time. It was noticed that on the sample without Rice ash, an absorption of 31.9% is observed after 28 days whereas 28.9% is observed after 90 days of curing time. On contrary, the addition of rice ash in the mix proportion has leaded to an improvement of the absorption. It was observed a slight reduction in water absorption on the samples without RA; this absorption has reached 8.36% of absorption after 28 days of curing and 1.79% of absorption after 90 days of curing. Similar to the impact seen on the limestone’s ability to withstand crushing, water absorption has produced consistent results after 28 days, 90 days, and with or without RA. This impact is caused by limestone’s lower mineral production, which leaves more open capillarity, which makes it easier for water to permeate. Limestone’s impact on water absorption is comparable to that of a sample taken without RA when compared to artificial aggregate that has been manufactured. This reaction can be attributed to the sample without RA having fewer minerals, which causes the pores in the sample to be covered.

#### 3.2.2. Softening Coefficient

**Figure 6:**
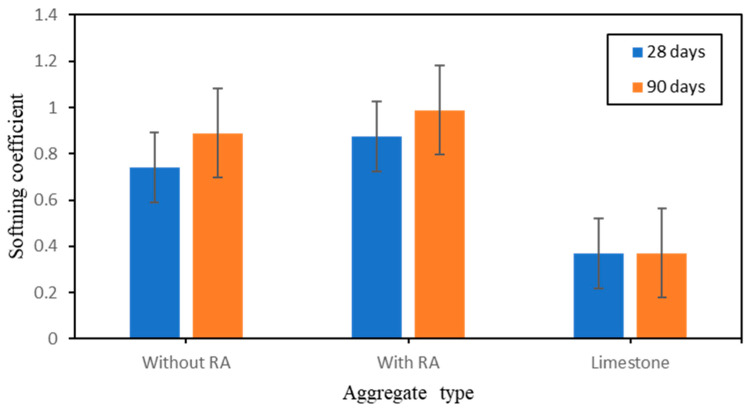
Effect of RA on the Softening behavior of aggregate types during 28 days and 90 days of curing time.

To characterize the effect of rice ash on the produced artificial aggregate based geopolymer, softening coefficient was determined to evaluate the water absorption properties. Figure 6 shows the results of the softening coefficient values. Basically, since each softening value are related to the crushing strength, the inter-relation shows correlated values. As the curing time increases, chemical reaction occurs and production of minerals increases hence the strength increases. This phenomenon has a direct influence on the softening value as, the higher the compressive strength the higher the softening coefficient value is and the lower the compressive strengthis, the lower the softening coefficient value is. Following this phenomena, the following softening coefficient values obtained after 28 days of curing and 90 days of curing has been noted: 0.74 and 0.89 without RA as well as 0.88 and 0.99 with addition of RA.

This effect shows a great correlation with the compressive strength values. It is also important to note that the density has a great impact and effect on both the compressive strength and the softening value. In this present work, the samples have not been heated in the oven hence, the samples did not reach required density. The fact that samples have not reached required density after curing time may affect the results of the compressive strength hence affect the result of the softening coefficient values due to the fact that the inner boundaries of the samples are still saturated. The limestone on the other hand have shown a low softening coefficient value of 0.37 compare to the produced artificial aggregates.

#### 3.2.3. Microhardness Value

**Figure 7:**
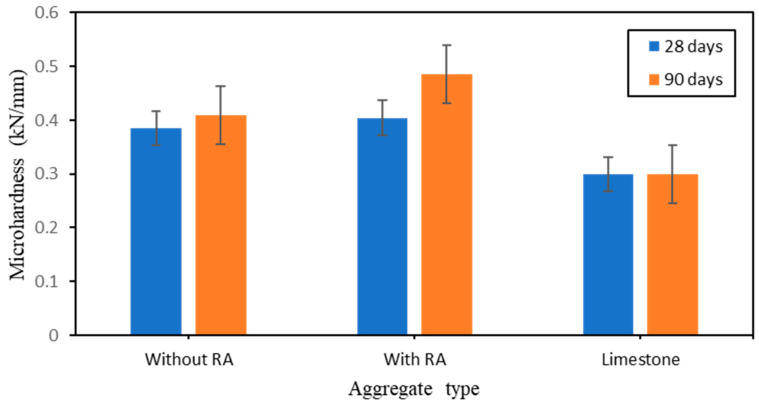
Effect of RA and PCA admixtures on the Microhardness of aggregate types.

The microhardness (Hv) value of sample surface is expressed in Figure 7. It shows that the development rules in Hv of the specimen surface with the addition of RA. In relation with artificial aggregates, the Hv value increase with the addition of RA. Moreover, the Hv increase as the curing time increase with the highest value of 0.49 kN/mm obtained after 90 days in contrast with the lowest value of 0.30 kN/mm obtained with the limestone. It is noticed that the Na_2_SiO_3_/NaOH mix ratio 1 leads to full balance of the mixture. Mortar mix ratio with Na_2_SiO_3_/NaOH mix ratios 1.0 have shown a good balance in the control of the mix proportion. It is important to specify that the addition of rice ash in the geopolymer based Fly Ash Alkaline has improved the strength of the Artificial Aggregate. Hence, rice ash can be used as sand replacement in the Artificial Aggregate mix proportion.

#### 3.2.4. Contact Angle and Adhesion

**Figure 8:**
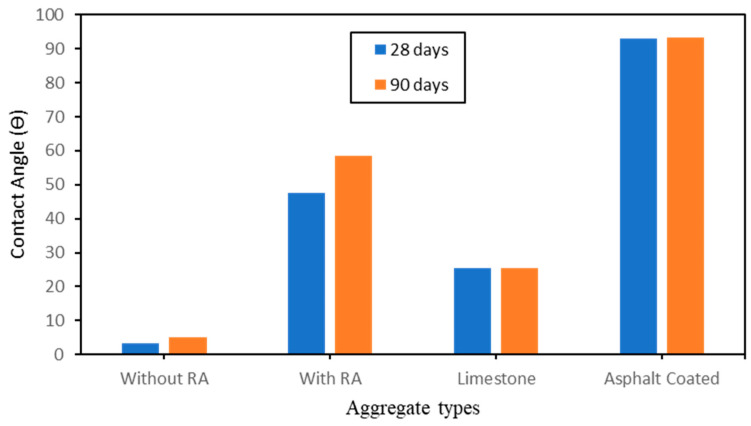
Effect of RA, Na_2_SiO_3/_NaOH and asphalt on the Contact Angle.

Contact angle constitutes another parameter defining the wettability of a material [26]. Figure 8 presents the results of the contact angles of water measured on each sample after curing times of 28 days and 90 days. As can be inferred from the obtained results, the nature of materials used for the mortar mixture has an impact on the values of water contact angles. The measurements of contact angles indicated that the specimens which were coated with asphalt (asphalt coated) is higher than the one of the produced artificial aggregates with and without RA similar to the limestone. This phenomenon can be understood by the fact that asphalt being a nature visco-elastic material, can easily cover all the existent pores present in the artificial aggregates hence, totally reduce the air-voids. On the other hand, it was noticed that the presence of RA in the mix proportion boosted the production of mineral compound groups which indirectly also cover the existent pores. A particular study and detail on the chemical characterization of the samples is given in this present work. Therefore, the use of both asphalt and high quantity of RA can leads to a full covering of existent pores present on the surface of the artificial aggregates and hence impact more beneficially on their hydrophobic nature. An important detail to note is that asphalt on one hand improves only the surface hydrophobic nature of the aggregates whereas rice ash on the other hand improves both the surface and the inner boundaries of the artificial aggregates. To be more specific the use of RA can lead to the production of amphiphile aggregates based geopolymer. In Figure 8, the contact angle increases simultaneously from 3.2° on the aggregate without RA after 28 days of curing time a value of 93.42° with addition of RA after 90 days of curing time. As specified above, the specimens coated with asphalt have showed a contact angle value of 93.10° after 28 days of curing and 93.42° after 90 days of curing; this showed a very short increase hence a really short impact of curing time with the use of asphalt. Moreover, the specimens with addition of RA have shown a contact angle value of 47.64° after 28 days of curing time and 58.64° after 90 days of curing time. This result shows that RA can slightly increase with time and hence produce more compact blocks facilitating the permeability of the specimens. The use of both activators’ sodium hydroxide and sodium silicate (Na_2_SiO_3_/NaOH) have also showed to influence the surface hydrophobic nature of the specimen.

**Figure 9:**
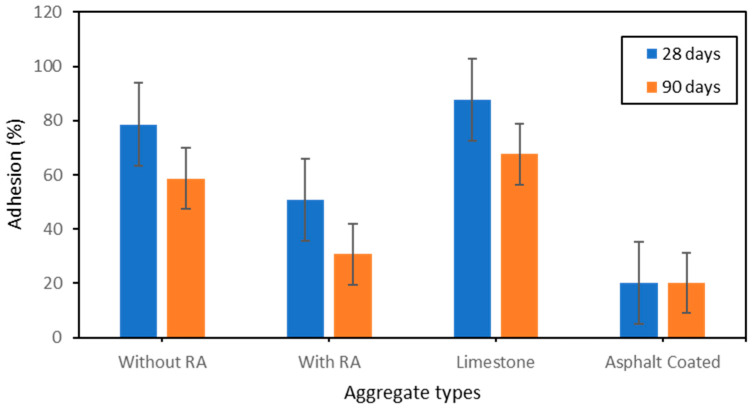
Effect of RA, and asphalt content on the adhesion after 28 days and 90 days curing time.

**Figure 10:**
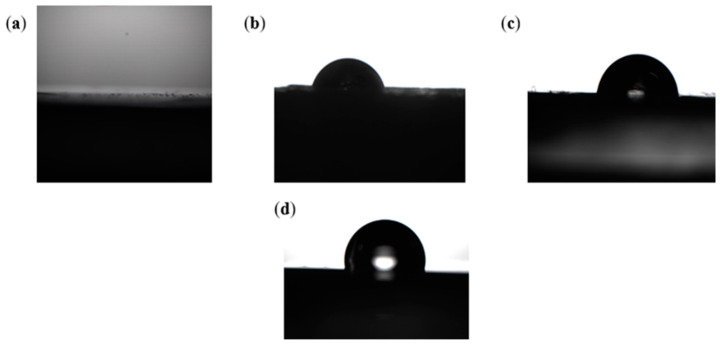
Artificial Aggregate surfaces during measurement of contact angle: (**a**) Limestone; (**b**) without RA; (**c**) with RA; (**d**) asphalt coated.

In addition to the contact angle, the adhesion between the aggregate and the liquid (water, asphalt, etc.) is another parameter to describe well how deep the absorption is, hence, how efficient is the aggregate in relation to it hydrophobic nature. Figure 9 depicts the results obtained from the adhesion test. From this figure (Figure 9), the same trend can be observed with the results showed in Figure 8; a good correlation is done here between the contact angle and the adhesion. It is observed that a change occurs in relation with curing time. The longer time the aggregate is in contact with natural air, the better it produces mineral blocks to prevent from high absorption. Aggregate coated with asphalt have shown to result with the best adhesion value. In the context of adhesion, the less the adhesion of the liquid is, the less the absorption is hence, the more hydrophobic the material is. After 28 days of curing time, it can be noted an adhesion of 20.2% with the aggregate coated with asphalt in contrast with an adhesion of 20.16% after 90 days of curing time. Similarly with the aggregate with addition of RA, without RA and limestone which have given adhesion values of 50.7%, 78.6% and 87.62% after 28 days of curing time in contrast with 30.7%, 58.6% and 67.62% after 90 days of curing time respectively to the order of type of aggregate. The reason of the obtention of these results can be due to the great effect of asphalt as coating material and also the good effect of both rice ash and alkaline solution (Na_2_SiO_3_/NaOH). Figure 10 depicts both the contact angle and the variation of the adhesion observed on each type of aggregate.

### 3.3. Morphology and Mineralogical Composition

#### 3.3.1. X-ray Diffraction (XRD)

**Figure 11:**
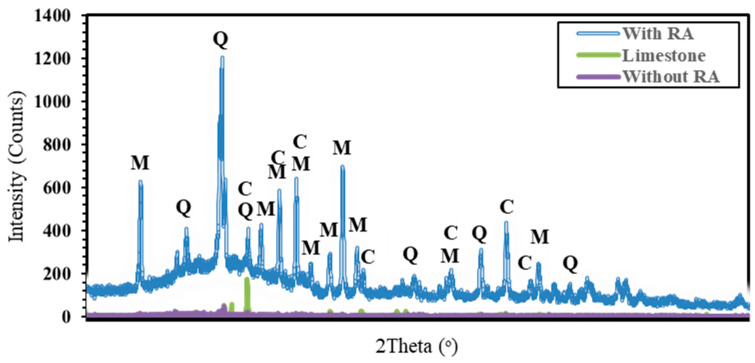
XRD patterns of different aggregate types (**M**: Mullite; **Q**: Quartz; **C**: Calcium silicate).

Figure 10 and Figure 11 shows both the mineralogical changes and the quantity of the different type of aggregate after 28 and 90 days of curing time. According to Figure 10 and Figure 11, it is noticed that quartz and mullites are the main crystallized products; none of these minerals can be observed in the limestone. The peak intensity of quartz phase enhanced with the addition of both RA and alkaline content, which is assigned to the introduction of silicon source, promoting the more formation of C-S-H gel and a long period time of hydration reaction. It also indicated that the more reaction production especially mullite formed with the addition of RA and good control of alkaline mix ratio, implying the reduction of CH due to the silicification of calcium sources. Just because of the introduction of abundant silicon source, the diffraction patterns of cristobalite enhance obviously due to the unreacted RA remains existent. In addition, the good amount of fly ash content in the design results in the peak intensity of both mullite and quartz which leads in the reduction of micro-porous groups hence, reduce voids.

**Figure 12:**
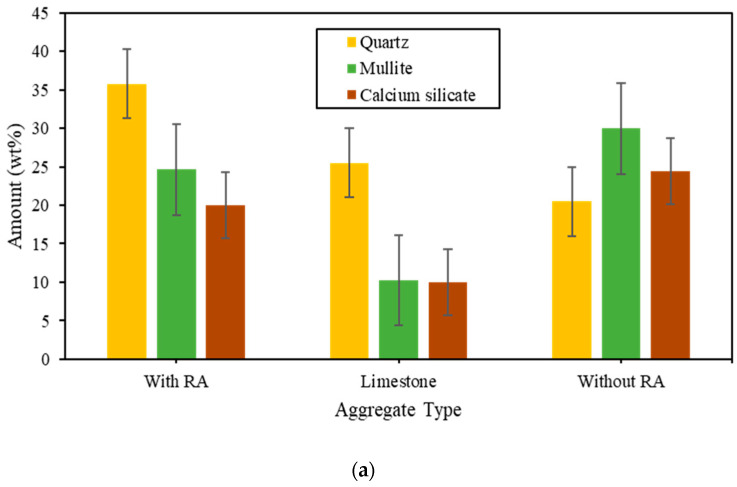

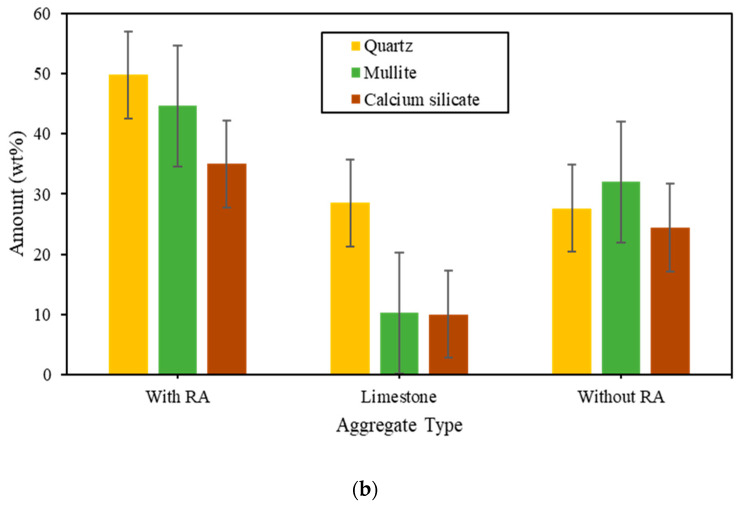
Quantity of mineral crystals produced after: (**a**) 28 days and (**b**) 90 days of curing time.

A good correlation can be done with the results obtained from Figure 5, Figure 6, Figure 7 and Figure 8 in addition to Figure 9 and Figure 10. Moreover, Calcium source materials with high degree of dissolution of calcium, silicon and aluminium have showed to provide high compressive strength. In addition, the alkalinity of the alkaline activator has also showed to be really beneficial in the enhancing of the compressive strength. The reaction is due to the presence of high quantity of RA in the mix proportion. Figure 12 have shown that the high presence of both sodium hydroxide and rice ash in the mix proportion leaded on one hand in the production of high amount of quartz and on another hand, in the presence of high amount of mullite which leaded to a perfect water resistance. Nonetheless, in comparison with the effect of asphalt on the water absorption of the produced artificial aggregates, it has been noticed that the amount of mullite was not high enough to reach a perfect hydrophobic nature.

#### 3.3.2. Scanning Electron Microscopy (SEM)

**Figure 13:**
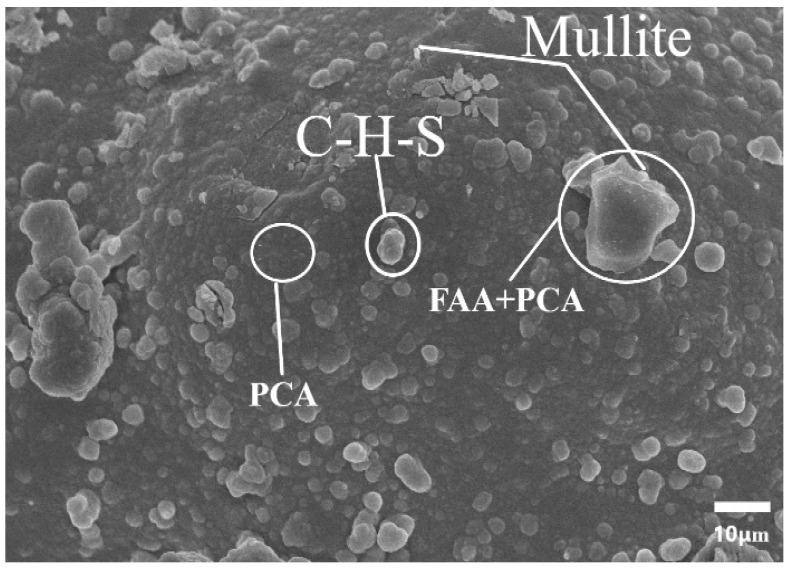
SEM image of the produced artificial aggregate.

In correlation with the results obtained from the XRD test, the artificial aggregate based geopolymer after curing times of 28 days and 90 days was chosen for SEM testing. Figure 13 shows the clear appearance of the produced principal minerals. Both the limestone and the aggregate without RA will appear a large number of voids and microcracks, which is caused by the lack of sufficient crystallization of the gel phase and function of micro-pores from hydration. As depicted in the Figure 13, the addition of RA shows the presence of C-S-H and mullite which gives a good correlation with the results presented from the XRD test on Figure 11. The role of rice as has an active filler and sand replacement in the sample can also be a good advantage. These phenomena persuasively testify that the proper addition of RA in the mix proportion is beneficial to the formation of a denser and homogeneous microstructure in geopolymer, consistent with the contact angle and microhardness results presented in this work.

### 3.4. Optimum Asphalt Binder Content

The aggregate source, binder type, and many other variables all influence the optimal binder content. The Fly ash employed in this study contains a fair amount of calcium, which provides anti-stripping qualities. The appropriate binder content depends on the amount of filler in the mixture and whether or not there are voids in the filler [27]. The volumetric properties of HMA are known to be influenced by the asphalt binder content, which is a vital parameter for ensuring the performance of the aggregate and, ultimately, the durability of the overall pavement construction. Higher binder content in a mix reduces compaction resistance since optimal binder content, which is reached after the entire mix design, also achieves maximum stability.

**Figure 14:**
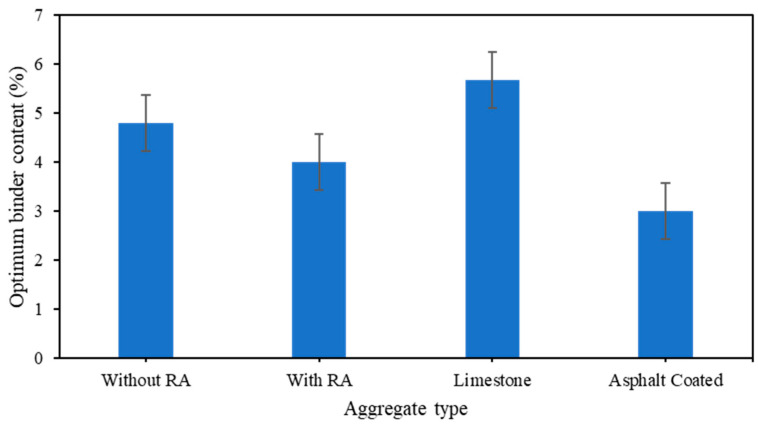
Different optimum binder contents in relationship to different type of mineral aggregates.

Since this study used activators to produce and better monitor the structure and mechanical properties of the produced artificial aggregate, more chemical interactions may have a potential impact on the required amount of asphalt binder for its good in-situ performance. The ideal asphalt binder has been identified based on the average values obtained from the primary graphs for the estimation of the optimal asphalt binder with Asphalt content vs. Density, Stability, Voids in Total Mixture (VTM), Voids Filled with Asphalt (VFA), and Flow(Figure 14). This result demonstrated that an optimal binder percentage of 4.2% should be used in asphalt concrete mixtures to properly employ this genuine new developed artificial aggregate as aggregate replacement; this result had a strong association with the findings from Figure 4 and Figure 7.

## 4. Conclusions

In this present work, a new artificial aggregate based geopolymer has been designed. The focused analyzed point in this work is the hydrophobic nature of the produced artificial aggregate based geopolymer improved on one hand with rice ash and on another hand coated with asphalt.

The following conclusions can be drawn:

(1) Na_2_SiO_3_/NaOH mix ratio 1 gives a better balance and control of the overall activating reaction.
(2) Sodium hydroxide (NaOH) is the solid part of the activators, hence, a quantity of 7.5–10% of alkali-silicate solution’s total weight leads to the reduction of pores.
(3) 90 days of curing has a great impact in both the compressive strength and the absorption; the longer time the aggregate is given to cure, the more solid mineral compound is produced.
(4) High quantity of rice ash can improve the compressive strength and, accelerate the production of mullite which is a great component for the hydrophobic nature of the aggregate.
(5) Asphalt coated aggregates improves the surface hydrophobicity of the artificial aggregate, hence, can be used as aggregate replacement in asphalt pavement mixtures.
(6) An optimum asphalt binder content ranges from 3–4.2% of asphalt content is required for a good wearing course and binder course coating.

## Figures and Tables

**Table 1:** Chemical Minerals Presents in the Alkali-Activated Aggregate and Limestone.

Chemical Formula	Mineral Crystals	Type of Aggregate (wt%)		
		With RA	Without RA	Limestone
SiO_2_	Quartz	27.64	43.6	49.76
3Al_2_O_3_·2SiO_2_	Mullite	44.64	31.98	10.24
Ca_2_O_4_Si	Calcium silicate	27.72	24.42	40

**Table 2:** Empirical Parameters of Asphalt.

Properties	Value	Method
Penetration @25 °C (dmm)	68	ASTM D 5-20 [18]
R&B Softening Point (°C)	49.2	ASTM D 36-14 [19]
Ductility @15 °C (cm)	158.4	ASTM D 113-17 [20]

**Table 3:** Step-by-step description of the experiment.

Description	Picture
Step 1: 15 cm × 15 cm × 15 cm cubic artificial aggregate based geopolymer specimen.	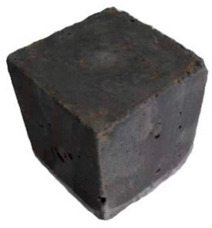
Step 2: crushing and sieving of specimens in different coarse and fine aggregate sizes in order to design the required aggregate gradation size.	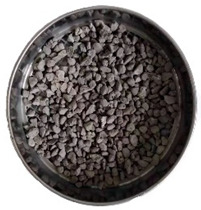
Step 3: coating of the sieved (coarse and fine) artificial aggregate based geopolymer with asphalt in order to reduce the pores present on the aggregates.	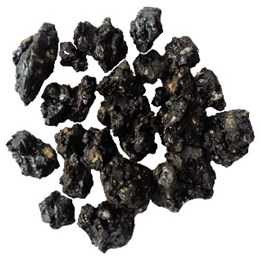
Step 4: use of the coated artificial aggregate based geopolymer with asphalt to make Marshall test specimens in order to determine the optimum asphalt binder content and hence, reduce the absorption of asphalt by the aggregates.	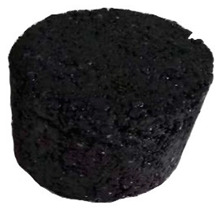

## Data Availability

Not applicable.
